# Recent Trends of Microbiota-Based Microbial Metabolites Metabolism in Liver Disease

**DOI:** 10.3389/fmed.2022.841281

**Published:** 2022-05-09

**Authors:** Raja Ganesan, Jin-Ju Jeong, Dong Joon Kim, Ki Tae Suk

**Affiliations:** Institute for Liver and Digestive Diseases, Hallym University, Chuncheon, South Korea

**Keywords:** microbial metabolomics, short-chain fatty acids, tryptophan metabolism, metabolic discrimination, liver therapies, metabolites alteration, liver diseases

## Abstract

The gut microbiome and microbial metabolomic influences on liver diseases and their diagnosis, prognosis, and treatment are still controversial. Research studies have provocatively claimed that the gut microbiome, metabolomics understanding, and microbial metabolite screening are key approaches to understanding liver cancer and liver diseases. An advance of logical innovations in metabolomics profiling, the metabolome inclusion, challenges, and the reproducibility of the investigations at every stage are devoted to this domain to link the common molecules across multiple liver diseases, such as fatty liver, hepatitis, and cirrhosis. These molecules are not immediately recognizable because of the huge underlying and synthetic variety present inside the liver cellular metabolome. This review focuses on microenvironmental metabolic stimuli in the gut-liver axis. Microbial small-molecule profiling (i.e., semiquantitative monitoring, metabolic discrimination, target profiling, and untargeted profiling) in biological fluids has been incompletely addressed. Here, we have reviewed the differential expression of the metabolome of short-chain fatty acids (SCFAs), tryptophan, one-carbon metabolism and bile acid, and the gut microbiota effects are summarized and discussed. We further present proof-of-evidence for gut microbiota-based metabolomics that manipulates the host's gut or liver microbes, mechanosensitive metabolite reactions and potential metabolic pathways. We conclude with a forward-looking perspective on future attention to the “dark matter” of the gut microbiota and microbial metabolomics.

## Introduction

The gut microbiome is a microbial ecosystem that has diverse effects on physiological metabolism, particularly microbial metabolic activity. The human gut microbiome is always changing. Many gastrointestinal metabolites are derived from dietary and environmental sources. Since a decade, the number of scientific publications on the gut microbiota has steadily increased. Gut microbiota-based metabolomics or metabolomics profiling examination has been proven to have the ability to screen and validate the metabolites' role in host and drug metabolism (1, 2). Clinical metabolomics profiling and chemical profiling from numerous host cells are used to evaluate a range of biological contexts at the level of metabolites or small molecules (low molecular weight, < 1500 Da) (3–7).

Clinical metabolomics has been advanced and placed as a division of systems biology. Gut microbiome-associated metabolites are directly connected with the liver via the portal vein. The gut microbiota directly yields the metabolome (full set of metabolites) and organic compounds (i.e., ethanol, acetaldehyde, ammonia, etc.). Metabolic compounds and bacterial products (pathogen-associated microbial metabolites) are frequently metabolized in liver cells (2, 8, 9).

The gut-liver pivot alludes to the multiple interactions between the intestinal microbiome and the liver, which can produce microbial metabolites. Microbial metabolic profiling acts as a therapeutic agent for specific liver diseases. These microbial profiling techniques play a significant role in heterogeneous liver diseases (10–12). At the point of planning an investigation and looking at proof, understanding the utility and impediments of both designated and untargeted metabolomics approaches are fundamental. Microbial metabolite evolution (additionally referred to as “clinical fluxomics”) can quantify and follow analytes through metabolic pathways (13, 14).

The metabolomics profiling in gut-microbiome and liver diseases have been rapidly growing since past decade. These data explain the importance of metabolomics research. The metabolome is inherently huge and complex. The non-targeted metabolome is more connected with the 16S rRNA microbiome composition than targeted metabolomics. This non-targeted metabolomics has identified novel metabolites in colorectal cancer (CRC) patients. High-throughput microbial community sequences have been studied (15, 16). Metabolites represent a functional change associated with genomic variation and differences in complex microbial communities. The microbial metabolites of SCFAs, such as butyrate, can influence gene expression, cell proliferation, and ultimately adenoma formation (17).

More interestingly, the microbial metabolic pathway-based human gut microbiome of monozygotic twins has been explained (18). Microbes such as *Escherichia coli* and *Saccharomyces cerevisiae* have 3,700 and 16,000 metabolites, respectively (19, 20).

Fundamental studies of various gut-organ axes are necessary in this domain. The metabotype (or metabolome) is basically different than the genotype. This metabotype designates what is happening in the cellular microenvironment. The genomics, transcriptomics, proteomics, metabolomics, and phenotype are now used in various areas in life science (21, 22). Untargeted and targeted metabolomics will transform what we source as medicine for every disease. Understanding of the microbiome and metabolome can be projected over the two decades.

## Gut Microbiota and Short Chain Fatty Acids

Gut microbiota-derived SCFAs (i.e., acetate, propionate, and butyrate) were found in the human large intestine and are involved in microbial fermentation (23–25). Table 1 shows that gut bacterial genera are involved in the fermentation process of SCFAs, amino acids, organic acids, polar metabolites, and dietary polyphenols. SCFAs play an important role in nutrients and energy from the intestinal epithelium. SCFAs are used for the maintenance of intestinal homeostasis. Various studies have confirmed that SCFAs participate in the regulation of NAFLD by activating G-protein-coupled receptor (GPR) 41 or 43, which are expressed in various areas, such as adipose, liver, tissues, peripheral blood, and intestinal cells (47, 48). As per previous publications, intestinal gluconeogenesis (IGN) functions as a regulator of NAFLD via upregulation of hepatic insulin sensitivity and downregulation of hepatic glucose production (HGP) through the gut-brain-liver neural circuit (49, 50).

**Table 1:** Host and microbial metabolic effects of SCFAs and other metabolite properties reported in recent literatures.

**Microbial source**	**Metabolites**	**Molecular Mass (Da)**	**Chemical formula**	**Physiological role**	**Ref**
*EHEC O157:H7* *Enterobacter sp*. *Bifidobacterium sp*.	Acetate (Acetic acid)	60.052	C_2_H_4_O_2_	Recovers gut barrier function	(26, 27)
*C. jejuni* *S. aureus*	Butyrate (Butyric acid)	88.11	C_4_H_8_O_2_	Recovers gut barrier function Decreases internalization; Rises the antimicrobial peptides	(25, 28) (29)
*Clostridium, Eubacterium, Faecalibacterium, Roseburia, and Butyrivibriocrossotus*				Pro-inflammatory studies -Anti-inflammatory expressions	(30)
*Coprococcus spp.Roseburia spp*.				Butyrate and acetate producers closely related to Ruminococcus.	(31)
*Anaerostipes* *caccae &* *Anaerostipes* *hadrus*				Butyrate producers, lactate and acetate utilizers.	(32, 33)
*S. aureus* *C. rodentium* *S. Typhimurium*	Propionate (Propionic acid)	74.08	C_3_H_6_O_2_	Decreases internalization; Increases antimicrobial peptides Enhances colonization	(34) (35)
				Intracellular pH stress	(36)
*S. aureus*	Hexanoate (Hexanoic acid or Caproic acid)	116.1583	C_6_H_12_O_2_	Decreases internalization; Increases antimicrobial peptides	(34)
*S. Typhimurium*	Butyrate	88.11	C_4_H_8_O_2_	Targets Salmonella pathogenicity island 1	(37, 38)
	(Butyric acid)			Acylation of transcriptional regulator attenuates virulence	(39)
				Targets Salmonella pathogenicity island 1	(38, 39)
				Inhibits oxygen availability	(40, 41)
				Inhibits translocation by inducing antimicrobial macrophage function	(42)
*S. Typhimurium* *Lactobacillus delbruekii* *Lactobacillus Jensenii*	Lactate (D-Lactic acid)	90.08	C_3_H_6_O_3_	Increases immune surveillance of mononuclear cells	(43)
*S. Typhimurium*	Pyruvate	88.06	C_3_H_3_O_3_	Increases immune surveillance of mononuclear cells	(43)
*C. jejuni* *Lactobacillus delbruekii* *Lactobacillus Jensenii*	Lactate (D-Lactic acid)	90.08	C_3_H_6_O_3_	Reduces virulence gene expression	(44)
*C. difficile*	Succinate	118.09	C_4_H_6_O_4_	Exacerbates infection	(45)
*EHEC O157:H7*				Enhances virulence gene expression	(46)

*C. jejuni, Campylobacter jejuni; S. aureus, Staphylococcus aureus; C. rodentium, Citrobacter rodentium; S. Typhimurium, Salmonella typhimurium; C. difficile, Clostridioides difficile; EHEC O157:H7, Escherichia coli O157:H7; Ref, References*.

**Figure 1:**
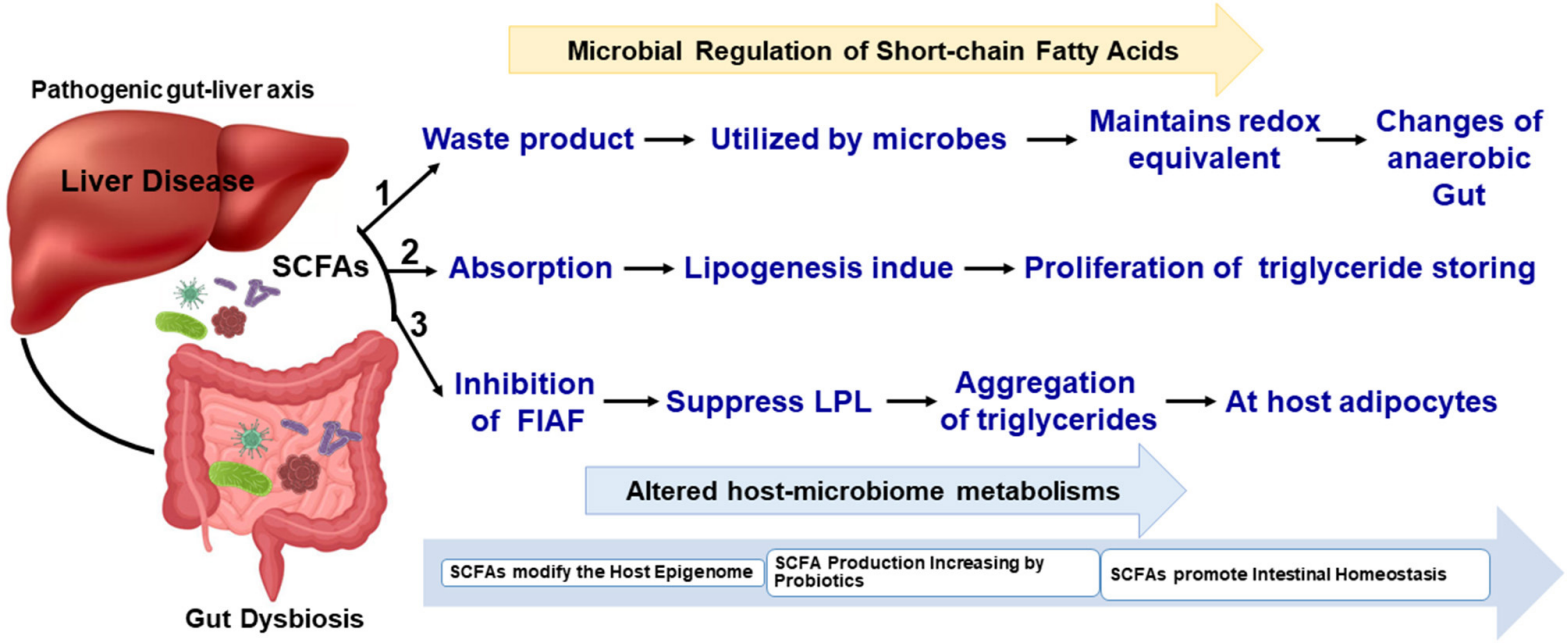
Simplified schematic view of SCFAs in the gut microbiome and liver metabolisms. The biochemical process and metabolites metabolisms have been connected with gut-liver microbiome interactions.

As shown in Table 1, the SCFAs acetate, propionate, hexanoate, pyruvate, lactate, succinate, and butyrate are significantly targeted IGNs, where glucose was *de novo* synthesized from the gut epithelium. Bacteria that belong to *Clostridium, Eubacterium, Faecalibacterium, Roseburia*, and *Butyrivibriocrossotus* has been producing butyrate through the reduction of two molecules of acetyl-CoA with synthesis of one molecule of ATP. These are most prominent butyrogenic bacteria groups. Studies reported the depletion of those bacteria in atherosclerosis. Butyrate is normally involved with preservation of the intestinal barrier function, tight junction proteins regulation and mucus layer maintenance. Here, glucose signaling to the brain via a GPR42-mediated neural circuit mechanism was widely initiated; therefore, glucose tolerance and insulin sensitivity were upregulated (51, 52). SCFAs are a product of bacterial fermentation of dietary fiber. According to protein sources, SCFAs can be formed by the gut microbiome (53). Figure 1 shows that the gut-liver axis is involved in SCFA alterations and their functional metabolism.

Additionally, GPR is initiated, and SCFAs mostly pass to the liver via the portal vein, where they can improve hepatic glycolipid homeostasis. This metabolic process initiation occurred via AMPK in a peroxisome proliferator-activated receptor (PPAR) γ-dependent manner (54). SCFAs travel across the blood–brain barrier (BBB) into the central nervous system (CNS) and can disturb neural development (neurogenesis, BBB permeability, microglia). The physiological process of gluconeogenesis, AMPK activity, and insulin sensitivity in the liver are significantly affected (52, 55). Finally, SCFAs are a significant signaling metabolome and are used for communication between host tissues and microbiota through the gut-brain-liver axis (56, 57).

## Gut Microbiota and Tryptophan Catabolites

The amino acid tryptophan exists in common foods (i.e., bananas, chocolate, cheese, fish, milk, oats, wine, etc.). Tryptophan is a chemically complex amino acid that can undergo an extensive variety of transformations within its structure (58, 59). Tryptophan acts as an ideal molecule in bacterial catabolic activity. To support this concept, various signaling pathways in human cells result from tryptophan, including tryptamine and serotonin. Dietary tryptophan is involved in numerous intermediates within hosts. The kynurenine and serotonin pathways are directly transformed from tryptophan. Protein synthesis occurs through the conversion of gut microbes into indole derivative metabolites such as indole acetic acid (IAA), indole-3-propionic acid (IPA), and indole-3-aldehyde (IA) (58, 60).

**Figure 2:**
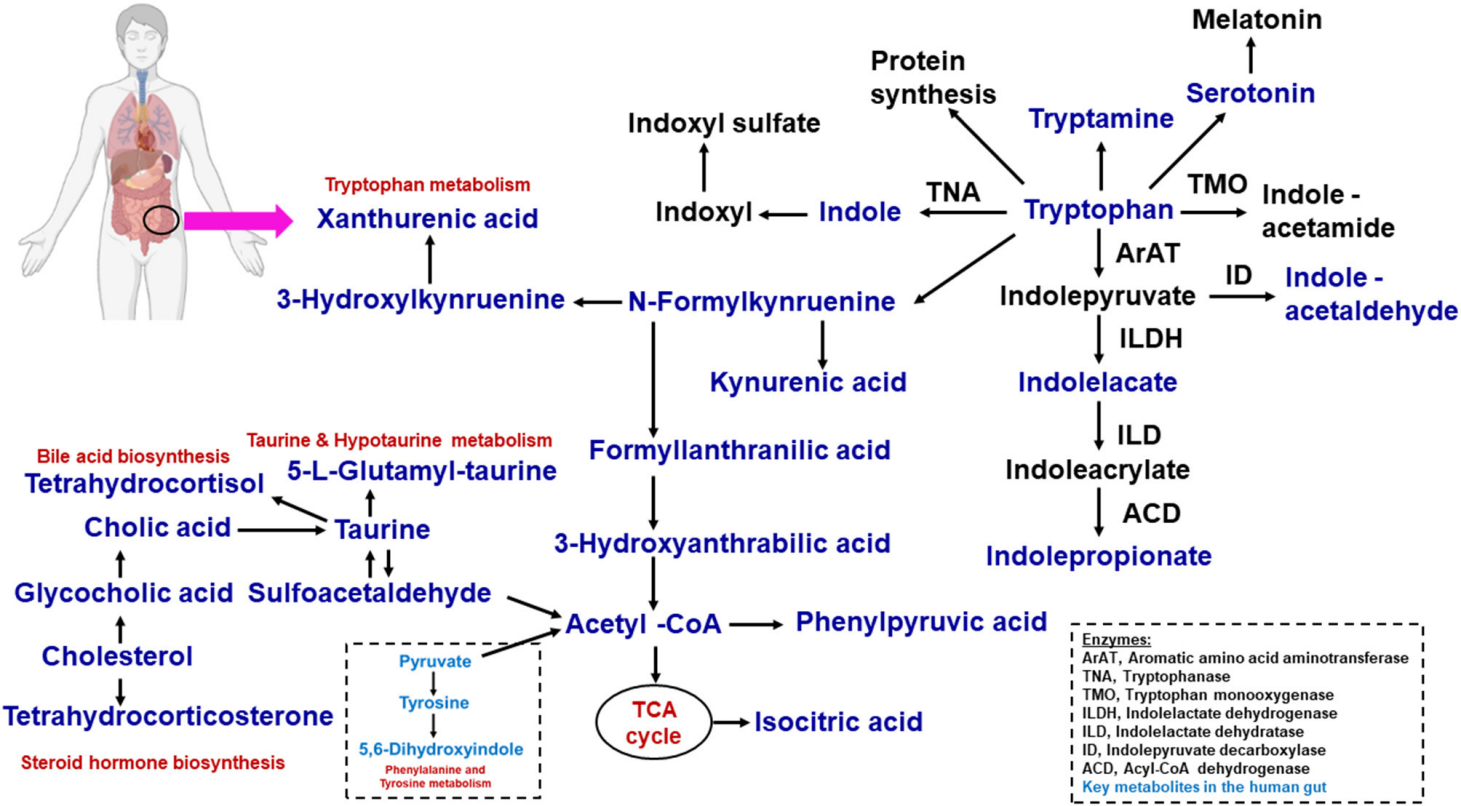
Microbial tryptophan is catabolized on host physiology. With the help of dietary proteins, the tryptophan is released by the gut microbiome. This tryptophan−1, the kynurenine pathway; 2, serotonin pathway; 3, protein synthesis; 4, direct transformation. The microbiome-associated metabolic relationship was identified from fatty acids, lipids, amino acids, and metabolites.

The kynurenine pathway contains many metabolic intermediates, collectively termed “kynurenines” and the final product, nicotinamide adenine dinucleotide (NAD+) (61, 62). The metabolic reaction of tryptophan to kynurenine is chemically converted to either indoleamine 2,3-dioxygenase 1 (IDO1, involved in immune and gut epithelial cells) or tryptophan 2,3-dioxygenase (TDO, hepatocytes) (62). The gut microbiota is a known driver of IDO1 expression (63, 64) and IDO1 regulation has been shown to regulate microbial community composition (65). These enzymes are highly increased in many cancer cells. Kynurenine derivatives are produced with aryl hydrocarbion receptor (AhR) ligands that help to promote cellular migration and immune tolerance, thus driving cancer progression (62). The host synthesizes kynurenines with the help of gut microbiota that have a genomic capacity to yield many intermediate small molecules in metabolic pathways, such as *Lactobacillus spp.*, and the pathogens *Pseudomonas aeruginosa* and *Pseudomonas fluorescens*, which produce these intermediates (66). We have listed in Table 2, Figure 2 the tryptophan metabolite-based microbiome and biological effects in human gut environments. Finally, kynurenine pathway intermediates significantly inhibited insulin synthesis, excretion, and signaling in rats. Increased levels of kynurenic acid and xanthurenic acid are found in type 2 diabetes mellitus (T2DM) patients (61).

**Table 2:** Examples of metabolic effects in host-microbial chemical transformation tryptophane family metabolites on pathogens.

**Microbial source**	**Metabolites**	**Molecular Mass (Da)**	**Chemical formula**	**Physiological role**	**Ref**
*P. aeruginosa* *S. Typhimurium* *C. albicans* *S. aureus* *V. cholerae* EHEC O157:H7	Indole	117.15	C_8_H_7_N	Increases biofilms; Decreases antimicrobials and virulence factors Increases multidrug resistance; Decreases motility and invasion genes Inhibits filamentation and biofilms Decreases regulatory and toxin gene expression Increases biofilms; Upregulates polysaccharide production Upregulates type III secretion system effectors	(67) (68, 69) (70) (71) (72) (73)
EHEC O157:H7	Indole-3- Aldehyde	145.156	C_9_H_7_NO	Inhibits filamentation and biofilms	(74)
*P. aeruginosa*	(Indole-3-carboxaldehyde)			Inhibits filamentation and biofilms	(74)
*C. albicans*				Upregulates IL-22 production by innate lymphoid cells	(65)
EHEC O157:H7	Indole-3 acetate	175.184	C_10_H_9_NO_2_	Inhibits biofilms, motility, and formation of lesions	(75)
EHEC O157:H7	7-hydroxyindole	133.15	C_8_H_7_NO	Inhibits biofilms	(76)
EHEC O157:H7	Skatole (3-methylindole)	131.172	C_9_H_9_N	Inhibits biofilms	(77)

*P. aeruginosa, Pseudomonas aeruginosa; S. Typhimurium, Salmonella typhimurium; C. albicans, Candida albicans; S. aureus, Staphylococcus aureus; V. cholerae, Vibrio cholerae; EHEC O157:H7, Escherichia coli O157:H7; Ref, References*.

Tryptamine and tryptophan catabolic chemical reactions are processed by gut microbial bacteria such as *C. sporogenes* and *Ruminococcus gnavus* (78, 79). Tryptamine acts as a β-arylamine neurotransmitter that can stimulate gut strength. In the gut microbial environment, tryptamine is known to induce the release of the neurotransmitter 5-hydroxytryptamine (5-HT) or serotonin via enterochromaffin cells, and it is involved in mucosal secretion and gut motility. 5-HT promotes gastrointestinal motility by acting on enteric nervous systems. However, the signaling molecule tryptamine affects the intestinal gut microbial composition, diversity, and metabolism in humans (80, 81). Here, ~90% of 5-HT or serotonin in the body is produced by enterochromaffin cells, which cannot cross the blood–brain barrier. The binding of 5-HT with specific 5-HT receptors produces various biological responses. In the central nervous system, 5-HT plays a central role in sleep, mood, appetite, behavior, and the maintenance of neurons and interstitial cells of Cajal within the gut myenteric plexus (80). In mice, *Ruminococcus flavefaciens* and *Adlercreutzia equolifaciens* reduced the beneficial properties of duloxetine. 5-HT is affected by the gut microbial composition, which acts as a gut microbial inhibitor (82, 83).

## Gut Microbiota and One Carbon Metabolisms

The membrane metabolite of choline acts as a water-soluble compound. This is an essential nutrient for human and animal cellular metabolism. Choline can contribute to cellular outer membrane functions, neurotransmission roles, and methyl donors for various biosynthetic metabolic reactions (84). Endogenously, choline is formed. Choline is widely used by anaerobic gut microorganisms to generate trimethylamine (TMA) and acetaldehyde (85). The gut microbiota plays an ameliorative role in liver diseases. Liver diseases such as fatty liver, hepatitis, and cirrhosis are related to bile acid secretion disorder and metabolic syndrome (56, 86, 87). Table 3 lists the more important metabolites in the human gut microbiome.

**Table 3:** Recent summary of host interaction and gut microbiome effects of significant amino acid metabolites on pathogens.

**Microbial source**	**Metabolites**	**Molecular Mass (Da)**	**Chemical formula**	**Physiological role**	**Ref**
*C. difficile*	Deoxycholate (Cholanoic acid)	392.572	C_24_H_40_O_4_	Prevents growth	(88–90)
*C. difficile*	Lithocholate (Lithocholic acid)	376.5726	C_24_H_40_O_3_	Prevents growth	(88–90)
*Influenza*	Desaminotyrosine (3-(4-Hydroxyphenyl)propionic acid)	166.17	C_9_H_10_O_3_	Upregulates type I interferons	(91)
*S. Typhimurium*	Vitamin B6 (Pyridoxine)	169.18	C_8_H_11_NO_3_	Encourages bacterial clearance	(92)
*S. aureus*	Vitamin B2 (Riboflavin)	376.36	C_17_H_20_N_4_O_6_	Shields against septic shock	(93)
*L. monocytogenes*				Upregulates antimicrobial agent	(94)
*S. aureus*	D-proline	115.13	C_5_H_9_NO_2_	Inhibits biofilms	(95)
*S. aureus*	D-tyrosine	181.19	C_9_H_11_NO_3_	Inhibits biofilms	(95)
*S. aureus*	D-phenylalanine	165.19	C_9_H_11_NO_2_	Inhibits biofilms	(95)
EHEC O157:H7	D-serine	105.09	C_3_H_7_NO_3_	Inhibits type III secretion system	(96)
*V. cholerae*	Trimethylamine	59.11	C_3_H_9_N	Overwhelms infection	(97)
*V. cholerae*	Cholic acid	408.57	C_24_H_40_O_5_	Overwhelms infection	(97)
*V. cholerae*	SCFAs			Overwhelms infection	(97)
*V. cholerae*	Several free D-amino acids			Increases antimicrobial H_2_O_2_	(98)

*C. difficile, Clostridioides difficile; S. Typhimurium, Salmonella typhimurium; S. aureus, Staphylococcus aureus; L. monocytogenes, Listeria monocytogenes; EHEC O157:H7, Escherichia coli O157:H7; V. cholerae, Vibrio cholerae; Ref, References*.

TMA is present across the host gut and can be processed to trimethylamine-N-oxide (TMAO) in the liver cellular microenvironment through flavin-containing monooxygenases 1 and 3 (FMO1 and FMO3). In the past few decades, gut-microbial-host cometabolites have been identified via metabolomics analysis in serum to predict the risk of cardiovascular diseases (99–101). TMAO enhances atherosclerosis by inducing multiple macrophage receptors, acting as a hallmark of thrombosis, and enhancing platelet reactivity (99, 100). Here, the gut microbiota facilitates the regulation of hepatic inflammation by the TMA, TMAO and FMO pathways.

Isotopic labeling studies have revealed that alterations of nutritional L-carnitine, a rich amino acid derived from red meat, increases TMA through microbiota-dependent conversion and leads to > 20-fold growth in atherogenic TMAO in omnivores vs. vegans and lactovegetarians (102). Recently, trimethyllysine (TML) was identified as a precursor to TMAO and could be used as a predictor of major adverse cardiac events. TML has improved risk stratification in acute coronary syndrome. TML is used to predict the risk of major adverse cardiac incidents and acts as a clinical biomarker for myocardial infarction (103, 104).

The B vitamins pyridoxine (vitamin B6), folic acid (vitamin B9) and cobalamin (vitamin B12) play essential roles in one-carbon metabolism. These vitamins act as cofactors in folate metabolism and one-carbon metabolic pathways. Moreover, B vitamins are not adequate in host synthesis to optimize metabolic conditions. B vitamins are also obtained from nutritional sources and *de nova* produced via the gut microbiota (105, 106). With the help of folate metabolism, the production of B vitamins by the colonic microbiota actually exceeds the dietary intake (107). Eight B vitamins (B1, B2, B3, B5, B6, B7, B9, and B12) have been discovered, and 40–65% of human gut bacteria have the genomic possibility to produce these vitamins. As per a prior database, 88% of vitamins in the gut microbiome were validated (106).

Folate metabolism (methotrexate and sulfasalazine) and genetic disorders very commonly occur due to B vitamin shortages and poor nutritional consumption. Pellagra (vitamin B3), anemias (vitamins B9 and B12), cerebellar ataxia (vitamin B12), and cognitive impairment (vitamins B9 and B12) have been linked with dietary deficiency that can be treated with vitamin supplementation. Here, there are age-dependent alterations in gut microbial metabolism of B vitamins (79, 108). An infant gut microbiome revealed that enriched genes could confuse the *de novo* biosynthesis of folate. The adult microbiome is enriched for those involved in the metabolism of folate and it is condensed from tetrahydrofolate (109, 110). Therefore, the gut microbiota is a fundamentally significant source for vitamin manufacture, which may be important for vitamin deficiencies. Finally, as shown in Table 4, metabolites and pathways associated with the gut microbiome in various liver diseases are summarized.

**Table 4:** Some examples of the most recently reported summary of gut microbiota-host interactions in various liver diseases-based metabolomics composition, and synthetic method.

**Metabolites**	**Metabolic pathways**	**Genera or species**	**Ref**
Phenylacetylglutamine (PAGln) and phenylacetylglycine (PAGly)	Synthesized during host hepatic phase II metabolism via conjugation of either glutamine or glycine to phenylacetic acid, an intermediate in microbial fermentation of phenylalanine	Conjugation of phenylacetic acid to glutamine or glycine occurs in the host liver; see *p-cresol* (above) for information about its precursor, phenylacetic acid	(3, 4)
Acetate (Acetic acid)	Pyruvate decarboxylation to acetyl-CoA	*Akkermansia muciniphila, Bacteroides spp., Bifidobacterium spp., Prevotella spp., Ruminococcus spp*.	(111–114)
	Wood–Ljungdahl pathway	*Blautia hydrogenotropphica, Clostridium* spp., *Streptococcus spp*.	(111–114)
Propionate (Propanoic acid)	Acrylate pathway	*Coprococcus catus, Eubacterium hallii, Megasphaera elsdenii, Veillonella spp*.	(111–114)
	Succinate pathway	*Bacteroides spp., Dialister spp., Phascolarctobacterium succinatutens, Veillonella spp*.	(111–114)
	Propanediol pathway	*Roseburia inulinivorans, Ruminococcus obeum, Salmonella enterica*.	(111–114)
Butyrate (Butanoic acid)	Classical pathway via butyrate kinase	*Coprococcus comes, Coprococcus eutactus*	(111–114)
	Alternate pathway using exogenous acetate	*Anaerostipes spp., C. catus, E. hallii, Eubacterium rectale, Faecalibacteerium prausnitzii, Roseburia spp*.	(111–114)
SCFAs and branched-chain fatty acids	Amino acid fermentation through various dissimilatory proteolytic reactions	*Acidaminococcus spp., Acidaminobacter spp., Campylobacter spp., Clostridia spp., Eubacterium spp., Fusobacterium spp., Peptostreptococcus spp*.	(112–115)
‘Kynurenines' (Kynurenine and its byproducts)	Many bacterial enzymes homologous to mammalian enzymes of the kynurenine pathway	*Lactobacillus spp., Pseudomonas aeruginosa, Putative: Pseudomonas spp., Xanthomonas spp., Burkholderia spp., Stenotrophomonas spp., Shewanella spp., Bacillus spp., members ofRhodobacteraceae, Micrococcaceae and Halomonadaceae families*	(66, 116)
Indole (Tryptophan metabolites)	Hydrolytic β-elimination of tryptophan to indole (tryptophanase)	*Achromobacter liquefaciens, Bacteroides ovatus, Bacteroides, thetaiotamicron, Escherichia coli, Paracolobactrum coliforme, Proteus vulgaris*	(116, 117).
Indole derivatives	Multiple	*Bacteroides spp., Clostridium spp. (Clostridium sporogenes, Clostridium cadaveris, Clostridium bartlettii), E. coli, Lactobacillus spp., E. halli, Parabacteroides distasonis, Peptostreptococcus spp. (Peptostreptococcus anaerobius)*	(4, 78, 116–119)
Tryptamine	Decarboxylation of tryptophan	*C. sporogenes, Ruminococcus gnavus*	(78)
Serotonin	Induction of host synthesis	*Indigenous spore-forming bacteria, dominated by Clostridium spp. and Turicibacter spp*.	(120, 121)
Histamine (Amino acid)	Decarboxylation of histidine (histidine decarboxylase: HDC)	*E. coli, Morganella morganii, Lactobacillus vaginalis* *Putative: Fusobacterium spp*.	(122, 123)
Imidazole propionate (ImP)	Non-oxidative deamination of histidine to urocanate followed by reduction of urocanate to ImP by urocanate reductase (UrdA)	*Aerococcus urinae, Adlercreutziae equolifaciens, Anaerococcus prevotii, Brevibacillus laterosporus, Eggerthella lenta, Lactobacillus paraplantarum, Shewanella oneidensis, Streptococcus mutans*	(124)
Dopamine	Decarboxylation of levodopa (l-DOPA) via tyrosine decarboxylase (TyrDC)	*Enterococcus spp. (Enterococcus faecalis, Enterococcus faecium, 77 human isolates of Enterococcus spp.), Lactobacillus brevis, Helicobacter pylori*	(125, 126)
*p*-Cresol	From tyrosine or phenylalanine via two pathways: direct cleavage of the Cα-Cβ bond in tyrosine to yield p-cresol by tyrosine lyase; and a series of reactions involving transamination, deamination and decarboxylation of tyrosine or phenylalanine via formation of the cresol precursor phenylacetic acid	*Assay proven: Blautia hydrogenotrophica, Clostridioides difficile, Olsenella uli, Romboutsia lituseburensis* *Predicted: Acidaminococcus fermentans, Anaerococcus vaginalis, Anaerostipes spp., Bacteroides spp., Bifidobacterium infantis, Blautia spp., Citrobacter koseri, Clostridium spp., Eubacterium siraeum, Fusobacterium spp., Klebsiella pneumoniae, Lactobacillus spp., M.elsdenii, Roseburia spp., Ruminococcus spp., Veillonella parvula*	(118)

*The microbial fermentation process depending on SCFAs, amino acids, organic acids, polar metabolites, and dietary polyphenols several liver diseases. HDC, Histidine decarboxylase; TyrDC, Tyrosine decarboxylase; Ref, References*.

## Gut Microbiome and Liver Ammonia Metabolism

Human liver is continually involved in ammonia detoxification. Ammonia fixation in the liver by glutamine and urea synthesis play main role in hepatic ammonia detoxification, and pH regulation under pathogenic condition. The liver and gut microbiota play a central role in nitrogen metabolism (127). Bacteria have involved in protein utilization and amino acid degradation. The understanding of bacteria process that carry out proteolysis and their following metabolic reactions is extremely relevant to human gut health. In large intestine, due to the protein catabolism, the toxic products of ammonia, indoles, and phenols were produced (128, 129). Amino acid fermentation has been primarily produced that the acetic, propionic, butyric, isobutyric, and isovaleric acid. From amino acid catabolism, the ammonia is constantly produced as a metabolic waste. The free ammonia is very toxic which rapidly converted to non-toxic urea via urea cycle in the liver and frequently excluded in urine (130). The liver can generate many enzymes which could change ammonia into urea (128). While ammonia level in blood becomes high, it may convert to toxic to brain. This condition is called as hyperammonemia (131). Hyper-ammonia producing ruminal bacteria (HAB) such as *Peptostreptococcus anaerobius, Clostridium sticklandii*, and *Clostridium aminophilum* has been involved to generate ammonia at high level (132). In this condition, the oxidation of ammonium to nitrite (${\text{NO}}_{2}^{-}$) with help of *Betaproteobacteria* and *Gammaproetobacteria* can happen by ammonia oxidizing bacteria (AOB). Liver failure and hepatocellular metabolic dysfunction can happen due to disturbed body nitrogen homeostasis. Due to the ammonia imbalance, hepatic encephalopathy is formed, which occurs when liver is high risk condition (133). Finally, chronic liver insufficiency is frequently associated with metabolic acidosis.

## Intestinal Microbiota and Bile Acid Metabolism

Bile acids (BAs) are important for cholesterol synthesis metabolism and fat breakdown and are synthesized from the liver and deposited in the gallbladder (134). In the small intestine, these BAs are secreted during digestion. BAs are reabsorbed in the terminal ileum by over 95% and returned to the liver by the portal vein. The absorption of directory fats, fat-soluble vitamins, and cholesterol is promoted by BAs (135). In addition, BAs act as signaling molecules that regulate glucose and lipid metabolism via farnesoid X receptor (FXR) activation and binding of G-protein coupled BA receptor 1 (136–138).

BAs are amphipathic molecules that influence intestinal mucosal integrity. The liver synthesizes BAs that are then involved in synthesizing antibacterial peptides, cholic acid, and chenodeoxycholic acid (139). Antimicrobial peptides (angiogenin 1) are formed when BAs bind to FXR. Activated FXR is involved in reducing the activity of the CYP7A1 gene through the nuclear receptor FXR. These peptides may inhibit intestinal mucosa overgrowth via the intestinal epithelial cell potential to block bacterial uptake, improving the gut barrier role (139). Most intestinal BAs are reabsorbed by the intestine, with 90–95% of BAs involved in enterohepatic circulation. The remaining BAs enter the colon, where the gut microbiome converts them into secondary and tertiary BAs (140, 141). Alterations in circulating BAs act as signaling molecules that disturb glucose and lipid metabolism and predispose individuals to NAFLD. The dysbiosis and disparity of BAs has been shown to play a significant role in liver disease control (141).

BAs are key signaling microbial metabolites involved in lesser-known axes. BAs are steroid acids, the conclusive end products of the liver cholesterol digestion system. There are four types of BAs: essential BAs, bile salts (or conjugated BAs), auxiliary BAs, and tertiary BAs. Essential BAs are liver-derived compounds and comprise a hydroxylated steroid center (142, 143). Cholic scarring and chenodeoxycholic corrosion could be caused by dysfunction of BAs. Bile salts are essential BAs that are conjugated with glycine or taurine (in people, higher primates, and rats) or taurine (in most other warm-blooded creatures) within liver metabolism (144).

**Figure 3:**
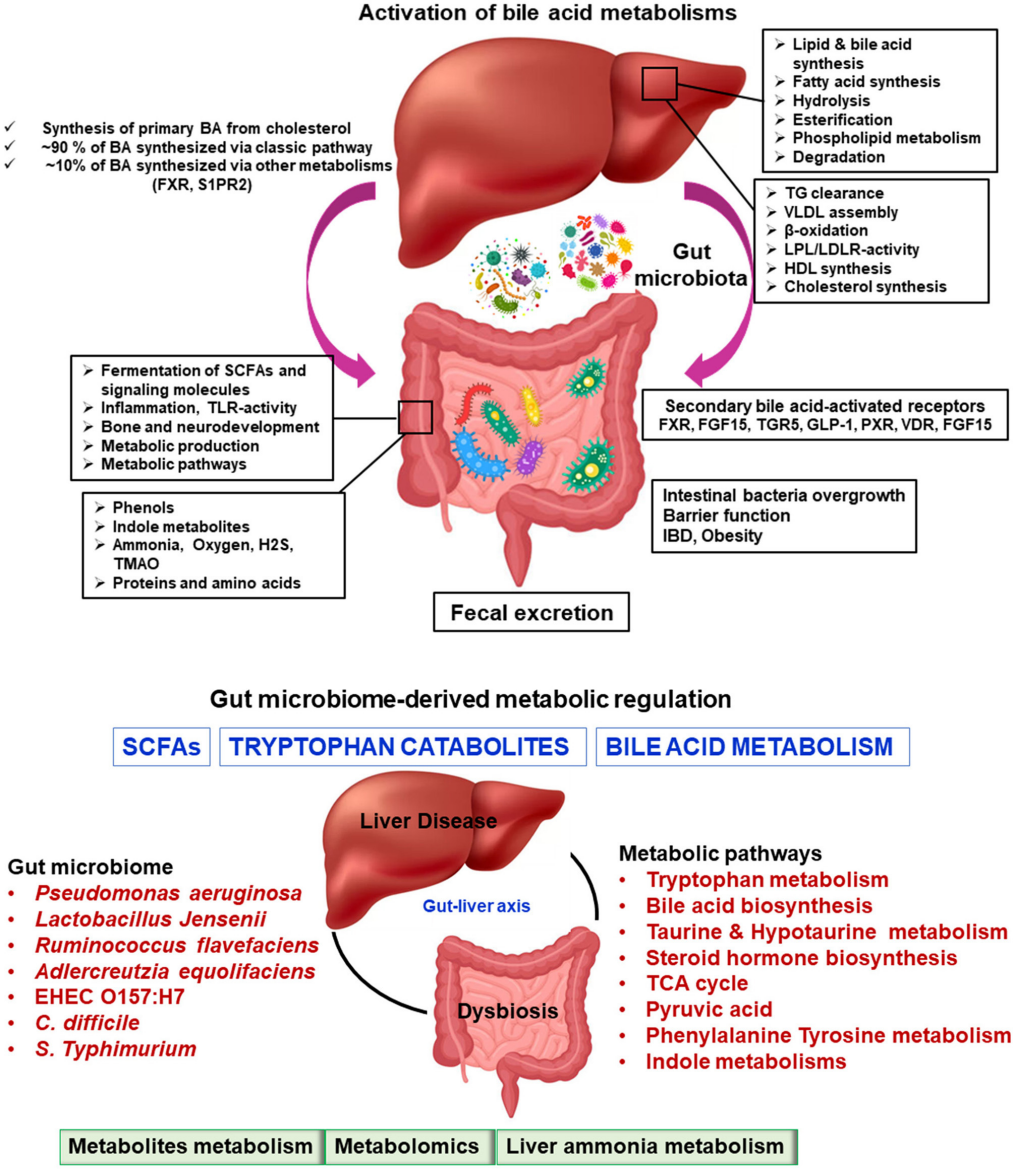
Microbiome-modulated metabolites and disease. Metabolite-based effects on liver disease process may be localized to the gastrointestinal tract which can influence to liver, heart, brain, etc. The summary of altered metabolic environments in gut-liver metabolic process.

These amino acid adjustments permit the bile salts to remain within the gently acidic pH of the upper portion of the little digestive tract. Auxiliary BAs are shaped by means of the activity of colonic microbes on bile salts, which remove the amino conjugates and assist in dihydroxylation of the parent compounds. This leads to the generation of compounds such as deoxycholic corrosive and lithocholic corrosive compounds (145–147). The more hydrophobic and hepatotoxic auxiliary BAs (such as lithocholate) may be altered by glucuronidation, hydroxylation, or sulfation to assist in their production. Tertiary BAs are shaped by the liver when bacterially created auxiliary keto-bile acids return to the liver and are degraded. For example, chenodeoxycholic corrosive (an essential bile salt) is converted to 7-ketolithocholic corrosive (an auxiliary bile corrosive) and then back to ursodeoxycholic corrosive (a tertiary bile corrosive) (148–150). Figure 3 summarizes the basic function of the liver and gut microbiome.

For metabolomic analysis, BA examination is a perfect reference metabolite (151). Naturally, there are more than 100 known BAs (i.e., essential BAs, auxiliary BAs, and tertiary BAs). Sensitive and multifold BA examinations imply quickly surveying a large number of BAs. This often results in a distinctly better understanding of the BA connections to one another and their individual physiological parts (152, 153). Whereas, metabolomic research on bile acids is providing new knowledge about human physiology and human pathologies, a few cautionary considerations must be kept in mind when studying BAs in non-human models. For example, rodents can hydroxylate bile acids at the 6-beta position (muricholates), whereas pigs can hydroxylate BAs at the 6-alpha position. As a result, discoveries with respect to the BA digestion system in animal models may not match those in people. The biological signaling of metabolites in the liver cellular microenvironment has a pleiotropic effect. These metabolites and the small-molecule metabolome are widely synthesized by the gut microbiota, as described in Tables 1–4.

In this review, we provided a fundamental overview of the gut microbiota and clinical metabolomics, including their history and recent developments, and future biomarker candidate metabolites. Currently, gut microbiota-associated metabolomics is in an early phase and needs to be more extensively researched. Identifying therapeutic biomarkers for various gut microbiome- and metabolome-based liver diseases are mandatory.

## Conclusions and Future Perspectives

In this review, we highlighted the SCFA, tryptophan, one-carbon metabolism, and bile acid metabolism in the gut microbiome from recent developments with the most promising microbial metabolites. The metabolites in the liver disease microenvironment provide deep knowledge of the metabolic pathways and microphysiological metabolism. The innovative approach of untargeted metabolomics is a quantitative method that is a unique, powerful new technology that can be combined with computational technologies. Improvements in the gut-liver metabolomics community, along with the continued effects of rapidly growing liver biology, have proven reasonably effective in understanding the gut-liver metabolic pathways and chemical reactions. Based on recent publications, the microbial metabolites of TMA, TMAO, tryptophan, SCFAs, vitamins, and the indole family have been found to come from the gut microbiota.

On the technology innovation front, we surveyed how gut microbiota and microbial metabolomics affect liver function and how to design and develop a clinical biomarker metabolite to protect the liver at the cellular microenvironmental level. The liver mechanisms and metabolic degradation analysis of the potential gut microbiome-associated liver metabolism are discussed. The key pitfall is still perhaps in the identification of microbial structural explanations of gut-liver metabolomics due to the lack of universal metabolite-specific libraries.

For the future perspective of gut-liver metabolomics in clinical biomarker development, we have realized a few thoughts:

1. The thickness and weight of liver tissue biopsy should be considered in the experimental design. To achieve a highly effective microbial metabolite, low-cost and facile modification methods should be used as much as possible. It is necessary to apply gut-liver metabolomics chemistry to expand their biological properties.
2. The solid liver tissue metabolome has a rich phenotypic response. This kind of metabolite production should be investigated further, including its processing and interfaces within the gut microbial environments.
3. Gut microbial metabolomics and metabolite chemical reactions at the microlevel need more attention in all gut-liver diseases. Defining how to address biochemical boundary communication in the liver tissue metabolome will help to better understand and optimize metabolomics functions at the gut-liver microcellular level.
4. The changes in molecular numbers should not be selectively mistreated for organic/inorganic metabolites in the gut microenvironment. High levels of change are a challenge when carrying out gut microbial metabolite-metabolite chemical reactions. Biological chemists are anticipated to develop novel microbial biomarkers/metabolites, tools, and materials for liver diseases with effective clinical applications.
5. Metagenomics, metabolomics, microbial metabolite profiling, and microbial small molecule screening are urgently needed to evaluate gut-liver disease properties. Moreover, more standard molecular, clinical and analytical measurement methods for gut-liver diseases are needed, which should be benchmarked.

## Author Contributions

RG wrote the manuscript draft, revised, and approved. J-JJ, DK, and KS participated in the revising the manuscript and approved. All authors contributed to the article and approved the submitted version.

## Funding

This research was supported by the Hallym University Research Fund and the Basic Science Research Program through the National Research Foundation (NRF) of Korea funded by the Ministry of Education, Science and Technology (NRF-2018M3A9F3020956, NRF-2019R1I1A3A01060447, NRF-2020R1I1A3073530, and NRF-2020R1A6A1A03043026).

## Conflict of Interest

The authors declare that they have no known conflicting financial interests or personal relationship that could have appeared to influence that work reported in this paper.

## Publisher's Note

All claims expressed in this article are solely those of the authors and do not necessarily represent those of their affiliated organizations, or those of the publisher, the editors and the reviewers. Any product that may be evaluated in this article, or claim that may be made by its manufacturer, is not guaranteed or endorsed by the publisher.

## References

[1] Bino RJ Hall RD Fiehn O Kopka J Saito K Draper J. Potential of metabolomics as a functional genomics tool. Trends Plant Sci. (2004) 9:418–25. 10.1016/j.tplants.2004.07.004 15337491

[2] Caporaso JG Kuczynski J Stombaugh J Bittinger K Bushman FD Costello EK. QIIME allows analysis of high-throughput community sequencing data. Nat Methods. (2010) 7:335–6. 10.1038/nmeth.f.303 20383131 PMC3156573

[3] Nemet I Saha PP Gupta N Zhu W Romano KA Skye SM. A cardiovascular disease-linked gut microbial metabolite acts via adrenergic receptors. Cell. (2020) 180:862–77. 10.1016/j.cell.2020.02.016 32142679 PMC7402401

[4] Dodd D Spitzer MH Van Treuren W Merrill BD Hryckowian AJ Higginbottom SK. A gut bacterial pathway metabolizes aromatic amino acids into nine circulating metabolites. Nature. (2017) 551:648–52. 10.1038/nature24661 29168502 PMC5850949

[5] Blow N. Metabolomics: Biochemistry's new look. Nature. (2008) 455:697–700. 10.1038/455697a 18833281

[6] Fessenden M. Metabolomics: small molecules, single cells. Nature. (2016) 540:153–5. 10.1038/540153a 27905420

[7] Holmes E Wilson ID Nicholson JK. metabolic phenotyping in health and disease. Cell. (2008) 134:714–7. 10.1016/j.cell.2008.08.026 18775301

[8] Miele L Marrone G Lauritano C Cefalo C Gasbarrini A Day C. Gut-liver axis and microbiota in NAFLD: insight pathophysiology for novel therapeutic target. Curr Pharm Des. (2013) 19:5314–24. 10.2174/1381612811319290011 23432669

[9] Raja G Jang Y-K Suh J-S Prabhakaran V-S Kim T-J. Advanced understanding of genetic risk and metabolite signatures in construction workers via cytogenetics and metabolomics analysis. Process Biochemistry. (2019) 86:117–26. 10.1016/j.procbio.2019.07.016

[10] Raja G Kim S Yoon D Yoon C Kim S. 1H NMR based metabolomics studies of the toxicity of titanium dioxide nanoparticles in zebrafish (Danio rerio). Bull Korean Chem Soc. (2018) 39:33–9. 10.1002/bkcs.11336

[11] Raja G Kim S Yoon D Yoon C Kim S. 1H-NMR-based metabolomics studies of the toxicity of mesoporous carbon nanoparticles in zebrafish (danio rerio). Bull Korean Chem Soc. (2017) 38:271–7. 10.1002/bkcs.11080

[12] Sumner LW Amberg A Barrett D Beale MH Beger R Daykin CA. Proposed minimum reporting standards for chemical analysis. Metabolomics. (2007) 3:211–21. 10.1007/s11306-007-0082-2 24039616 PMC3772505

[13] Srivastava A Kowalski GM Callahan DL Meikle PJ Creek DJ. Strategies for extending metabolomics studies with stable isotope labelling and fluxomics. Metabolites. (2016) 6:32. 10.3390/metabo6040032 27706078 PMC5192438

[14] Zamboni N Saghatelian A Patti GJ. Defining the metabolome: size, flux, and regulation. Mol Cell. (2015) 58:699–706. 10.1016/j.molcel.2015.04.021 26000853 PMC4831058

[15] Melnik AV da Silva RR Hyde ER Aksenov AA Vargas F Bouslimani A. Coupling targeted and untargeted mass spectrometry for metabolome-microbiome-wide association studies of human fecal samples. Anal Chem. (2017) 89:7549–59. 10.1021/acs.analchem.7b01381 28628333

[16] Caporaso JG Lauber CL Walters WA Berg-Lyons D Huntley J Fierer N. Ultra-high-throughput microbial community analysis on the Illumina HiSeq and MiSeq platforms. ISME J. (2012) 6:1621–4. 10.1038/ismej.2012.8 22402401 PMC3400413

[17] Bultman SJ Jobin C. Microbial-derived butyrate: an oncometabolite or tumor-suppressive metabolite? Cell Host Microbe. (2014) 16:143–5. 10.1016/j.chom.2014.07.011 25121740 PMC4179296

[18] Turnbaugh PJ Hamady M Yatsunenko T Cantarel BL Duncan A Ley RE. A core gut microbiome in obese and lean twins. Nature. (2009) 457:480–4. 10.1038/nature07540 19043404 PMC2677729

[19] Ramirez-Gaona M Marcu A Pon A Guo AC Sajed T Wishart NA. YMDB 2. 0: a significantly expanded version of the yeast metabolome database. Nucleic Acids Res. (2017) 45:D440–5. 10.1093/nar/gkw1058 27899612 PMC5210545

[20] Sajed T Marcu A Ramirez M Pon A Guo AC Knox C. ECMDB 2. 0: A richer resource for understanding the biochemistry of E coli. Nucleic acids research. (2016) 44:D495–501. 10.1093/nar/gkv1060 26481353 PMC4702790

[21] Xia J Sinelnikov IV Han B Wishart DS. MetaboAnalyst 3. 0—making metabolomics more meaningful. Nucleic Acids Research. (2015) 43:W251–7. 10.1093/nar/gkv380 25897128 PMC4489235

[22] Wishart DS Sykes BD Richards FM. The chemical shift index: a fast and simple method for the assignment of protein secondary structure through NMR spectroscopy. Biochemistry. (1992) 31:1647–51. 10.1021/bi00121a010 1737021

[23] Hu J Lin S Zheng B Cheung PCK. Short-chain fatty acids in control of energy metabolism. Crit Rev Food Sci Nutr. (2018) 58:1243–9. 10.1080/10408398.2016.1245650 27786539

[24] Hoyles L Fernández-Real JM Federici M Serino M Abbott J Charpentier J. Molecular phenomics and metagenomics of hepatic steatosis in non-diabetic obese women. Nat Med. (2018) 24:1070–80. 10.1038/s41591-018-0061-3 29942096 PMC6140997

[25] Nicholson JK Holmes E Kinross J Burcelin R Gibson G Jia W. Host-gut microbiota metabolic interactions. Science. (2012) 336:1262–7. 10.1126/science.1223813 22674330

[26] Fukuda S Toh H Hase K Oshima K Nakanishi Y Yoshimura K. Bifidobacteria can protect from enteropathogenic infection through production of acetate. Nature. (2011) 469:543–7. 10.1038/nature09646 21270894

[27] Fukuda S Toh H Taylor TD Ohno H Hattori M. Acetate-producing bifidobacteria protect the host from enteropathogenic infection via carbohydrate transporters. Gut Microbes. (2012) 3:449–54. 10.4161/gmic.21214 22825494

[28] Van Deun K Pasmans F Van Immerseel F Ducatelle R Haesebrouck F. Butyrate protects Caco-2 cells from Campylobacter jejuni invasion and translocation. Br J Nutr. (2008) 100:480–4. 10.1017/S0007114508921693 18275629

[29] Ochoa-Zarzosa A Villarreal-Fernández E Cano-Camacho H López-Meza JE. Sodium butyrate inhibits Staphylococcus aureus internalization in bovine mammary epithelial cells and induces the expression of antimicrobial peptide genes. Microb Pathog. (2009) 47:1–7. 10.1016/j.micpath.2009.04.006 19393738

[30] Zhang S-L Wang S-N Miao C-Y. Influence of microbiota on intestinal immune system in ulcerative colitis and its intervention. Front Immunol. (2017) 8:1674. 10.3389/fimmu.2017.01674 29234327 PMC5712343

[31] Louis P Flint HJ. Diversity, metabolism and microbial ecology of butyrate-producing bacteria from the human large intestine. FEMS Microbiol Lett. (2009) 294:1–8. 10.1111/j.1574-6968.2009.01514.x 19222573

[32] Duncan SH Louis P Flint HJ. Lactate-utilizing bacteria, isolated from human feces, that produce butyrate as a major fermentation product. Appl Environ Microbiol. (2004) 70:5810–7. 10.1128/AEM.70.10.5810-5817.2004 15466518 PMC522113

[33] Allen-Vercoe E Daigneault M White A Panaccione R Duncan SH Flint HJ. Anaerostipes hadrus comb. nov., a dominant species within the human colonic microbiota; reclassification of Eubacterium hadrum Moore et al. 1976. Anaerobe. (2012) 18:523–9. 10.1016/j.anaerobe.2012.09.002 22982042

[34] Alva-Murillo N Ochoa-Zarzosa A López-Meza JE. Short chain fatty acids (propionic and hexanoic) decrease Staphylococcus aureus internalization into bovine mammary epithelial cells and modulate antimicrobial peptide expression. Vet Microbiol. (2012) 155:324–31. 10.1016/j.vetmic.2011.08.025 21930351

[35] Connolly JPR Slater SL O'Boyle N Goldstone RJ Crepin VF Ruano-Gallego D. Host-associated niche metabolism controls enteric infection through fine-tuning the regulation of type 3 secretion. Nat Commun. (2018) 9:4187. 10.1038/s41467-018-06701-4 30305622 PMC6180029

[36] Jacobson A Lam L Rajendram M Tamburini F Honeycutt J Pham T. A gut commensal-produced metabolite mediates colonization resistance to salmonella infection. Cell Host Microbe. (2018) 24:296–307. 10.1016/j.chom.2018.07.002 30057174 PMC6223613

[37] Hung CC Garner CD Slauch JM Dwyer ZW Lawhon SD Frye JG. The intestinal fatty acid propionate inhibits Salmonella invasion through the post-translational control of HilD. Mol Microbiol. (2013) 87:1045–60. 10.1111/mmi.12149 23289537 PMC3581741

[38] Lawhon SD Maurer R Suyemoto M Altier C. Intestinal short-chain fatty acids alter Salmonella typhimurium invasion gene expression and virulence through BarA/SirA. Mol Microbiol. (2002) 46:1451–64. 10.1046/j.1365-2958.2002.03268.x 12453229

[39] Zhang ZJ Pedicord VA Peng T Hang HC. Site-specific acylation of a bacterial virulence regulator attenuates infection. Nat Chem Biol. (2020) 16:95–103. 10.1038/s41589-019-0392-5 31740807 PMC8439376

[40] Rivera-Chávez F Zhang LF Faber F Lopez CA Byndloss MX Olsan EE. Depletion of butyrate-producing clostridia from the gut microbiota drives an aerobic luminal expansion of salmonella. Cell Host Microbe. (2016) 19:443–54. 10.1016/j.chom.2016.03.004 27078066 PMC4832419

[41] Byndloss MX Olsan EE Rivera-Chávez F Tiffany CR Cevallos SA Lokken KL. Microbiota-activated PPAR-γ signaling inhibits dysbiotic Enterobacteriaceae expansion. Science. (2017) 357:570–5. 10.1126/science.aam9949 28798125 PMC5642957

[42] Schulthess J Pandey S Capitani M Rue-Albrecht KC Arnold I Franchini F. The Short chain fatty acid butyrate imprints an antimicrobial program in macrophages. Immunity. (2019) 50:432–45. 10.1016/j.immuni.2018.12.018 30683619 PMC6382411

[43] Morita N Umemoto E Fujita S Hayashi A Kikuta J Kimura I. GPR31-dependent dendrite protrusion of intestinal CX3CR1(+) cells by bacterial metabolites. Nature. (2019) 566:110–4. 10.1038/s41586-019-0884-1 30675063

[44] Luethy PM Huynh S Ribardo DA Winter SE Parker CT Hendrixson DR. Microbiota-derived short-chain fatty acids modulate expression of campylobacter jejuni determinants required for commensalism and virulence. m*Bio*. (2017) 8:e00407-17. 10.1128/mBio.00407-17 28487428 PMC5424204

[45] Ferreyra JA Wu KJ Hryckowian AJ Bouley DM Weimer BC Sonnenburg JL. Gut microbiota-produced succinate promotes C. difficile infection after antibiotic treatment or motility disturbance. Cell Host Microbe. (2014) 16:770–7. 10.1016/j.chom.2014.11.003 25498344 PMC4859344

[46] Curtis MM Hu Z Klimko C Narayanan S Deberardinis R Sperandio V. The gut commensal Bacteroides thetaiotaomicron exacerbates enteric infection through modification of the metabolic landscape. Cell Host Microbe. (2014) 16:759–69. 10.1016/j.chom.2014.11.005 25498343 PMC4269104

[47] Kimura I Inoue D Hirano K Tsujimoto G. The SCFA receptor GPR43 and energy metabolism. Front Endocrinol. (2014) 5:85. 10.3389/fendo.2014.00085 24926285 PMC4046487

[48] Inoue D Tsujimoto G Kimura I. Regulation of energy homeostasis by GPR41. Front Endocrinol. (2014) 5:81. 10.3389/fendo.2014.00081 24904531 PMC4033597

[49] Fujii H Kawada N. NAFLD JSGo. The role of insulin resistance and diabetes in nonalcoholic fatty liver disease. Int J Mol Sci. (2020) 21:3863. 10.3390/ijms21113863 32485838 PMC7312931

[50] Barataud A Vily-Petit J Goncalves D Zitoun C Duchampt A Philippe E. Metabolic benefits of gastric bypass surgery in the mouse: the role of fecal losses. Mol Metab. (2020) 31:14–23. 10.1016/j.molmet.2019.11.006 31918916 PMC6880100

[51] de Vadder F Mithieux G. Gut-brain signaling in energy homeostasis: the unexpected role of microbiota-derived succinate. J Endocrinol. (2018) 236:R105–r8. 10.1530/JOE-17-0542 29321189

[52] Koh A De Vadder F Kovatcheva-Datchary P Bäckhed F. From dietary fiber to host physiology: short-chain fatty acids as key bacterial metabolites. Cell. (2016) 165:1332–45. 10.1016/j.cell.2016.05.041 27259147

[53] Blacher E Levy M Tatirovsky E Elinav E. Microbiome-modulated metabolites at the interface of host immunity. J Immunol. (2017) 198:572–80. 10.4049/jimmunol.1601247 28069752

[54] den Besten G Bleeker A Gerding A van Eunen K Havinga R van Dijk TH. Short-chain fatty acids protect against high-fat diet-induced obesity via a PPARγ-dependent switch from lipogenesis to fat oxidation. Diabetes. (2015) 64:2398–408. 10.2337/db14-1213 25695945

[55] Mitchell RW On On NH Del Bigio MR Miller DW Hatch GM. Fatty acid transport protein expression in human brain and potential role in fatty acid transport across human brain microvessel endothelial cells. J Neurochem. (2011) 117:735–46. 10.1111/j.1471-4159.2011.07245.x 21395585

[56] Eom JA Kwon GH Kim NY Park EJ Won SM Jeong JJ. Diet-regulating microbiota and host immune system in liver disease. Int J Mol Sci. (2021) 22:6326. 10.3390/ijms22126326 34199182 PMC8231888

[57] Gebru YA Choi MR Raja G Gupta H Sharma SP Choi YR. Pathophysiological roles of mucosal-associated invariant T cells in the context of gut microbiota-liver axis. Microorganisms. (2021) 9:296. 10.3390/microorganisms9020296 33535703 PMC7912788

[58] Alkhalaf LM Ryan KS. Biosynthetic manipulation of tryptophan in bacteria: pathways and mechanisms. Chem Biol. (2015) 22:317–28. 10.1016/j.chembiol.2015.02.005 25794436

[59] Roager HM Licht TR. Microbial tryptophan catabolites in health and disease. Nat Commun. (2018) 9:3294. 10.1038/s41467-018-05470-4 30120222 PMC6098093

[60] Raja G Gupta H Gebru YA Youn GS Choi YR Kim HS. Recent advances of microbiome-associated metabolomics profiling in liver disease: principles, mechanisms, and applications. Int J Mol Sci. (2021) 22:1160. 10.3390/ijms22031160 33503844 PMC7865944

[61] Cervenka I Agudelo LZ Ruas JL. Kynurenines: Tryptophan's metabolites in exercise, inflammation, and mental health. Science. (2017) 357:eaaf9794. 10.1126/science.aaf9794 28751584

[62] Houtkooper RH Cantó C Wanders RJ Auwerx J. The secret life of NAD+: an old metabolite controlling new metabolic signaling pathways. Endocr Rev. (2010) 31:194–223. 10.1210/er.2009-0026 20007326 PMC2852209

[63] Atarashi K Tanoue T Shima T Imaoka A Kuwahara T Momose Y. Induction of colonic regulatory T cells by indigenous Clostridium species. Science. (2011) 331:337–41. 10.1126/science.1198469 21205640 PMC3969237

[64] Rhee SJ Walker WA Cherayil BJ. Developmentally regulated intestinal expression of IFN-gamma and its target genes and the age-specific response to enteric Salmonella infection. J Immunol. (2005) 175:1127–36. 10.4049/jimmunol.175.2.1127 16002714

[65] Zelante T Iannitti RG Cunha C De Luca A Giovannini G Pieraccini G. Tryptophan catabolites from microbiota engage aryl hydrocarbon receptor and balance mucosal reactivity via interleukin-22. Immunity. (2013) 39:372–85. 10.1016/j.immuni.2013.08.003 23973224

[66] Vujkovic-Cvijin I Dunham RM Iwai S Maher MC Albright RG Broadhurst MJ. Dysbiosis of the gut microbiota is associated with HIV disease progression and tryptophan catabolism. Sci Transl Med. (2013) 5:193ra91. 10.1126/scitranslmed.3006438 23843452 PMC4094294

[67] Lee J Attila C Cirillo SL Cirillo JD Wood TK. Indole and 7-hydroxyindole diminish Pseudomonas aeruginosa virulence. Microb Biotechnol. (2009) 2:75–90. 10.1111/j.1751-7915.2008.00061.x 21261883 PMC3815423

[68] Nikaido E Giraud E Baucheron S Yamasaki S Wiedemann A Okamoto K. Effects of indole on drug resistance and virulence of Salmonella enterica serovar Typhimurium revealed by genome-wide analyses. Gut Pathog. (2012) 4:5. 10.1186/1757-4749-4-5 22632036 PMC3405474

[69] Kohli N Crisp Z Riordan R Li M Alaniz RC Jayaraman A. The microbiota metabolite indole inhibits Salmonella virulence: Involvement of the PhoPQ two-component system. PLoS ONE. (2018) 13:e0190613. 10.1371/journal.pone.0190613 29342189 PMC5771565

[70] Oh S Go GW Mylonakis E Kim Y. The bacterial signalling molecule indole attenuates the virulence of the fungal pathogen Candida albicans. J Appl Microbiol. (2012) 113:622–8. 10.1111/j.1365-2672.2012.05372.x 22726313

[71] Lee JH Cho HS Kim Y Kim JA Banskota S Cho MH. Indole and 7-benzyloxyindole attenuate the virulence of Staphylococcus aureus. Appl Microbiol Biotechnol. (2013) 97:4543–52. 10.1007/s00253-012-4674-z 23318836

[72] Mueller RS Beyhan S Saini SG Yildiz FH Bartlett DH. Indole acts as an extracellular cue regulating gene expression in vibrio cholerae. J Bacteriol. (2009) 191:3504–16. 10.1128/JB.01240-08 19329638 PMC2681914

[73] Hirakawa H Kodama T Takumi-Kobayashi A Honda T Yamaguchi A. Secreted indole serves as a signal for expression of type III secretion system translocators in enterohaemorrhagic Escherichia coli O157:H7. Microbiology. (2009) 155:541–50. 10.1099/mic.0.020420-0 19202102

[74] Lee JH Cho MH Lee J. 3-indolylacetonitrile decreases Escherichia coli O157:H7 biofilm formation and Pseudomonas aeruginosa virulence. Environ Microbiol. (2011) 13:62–73. 10.1111/j.1462-2920.2010.02308.x 20649646

[75] Bommarius B Anyanful A Izrayelit Y Bhatt S Cartwright E Wang W. A family of indoles regulate virulence and shiga toxin production in pathogenic E. coli. PLOS ONE. (2013) 8:e54456. 10.1371/journal.pone.0054456 23372726 PMC3553163

[76] Lee J Bansal T Jayaraman A Bentley WE Wood TK. Enterohemorrhagic Escherichia coli biofilms are inhibited by 7-hydroxyindole and stimulated by isatin. Appl Environ Microbiol. (2007) 73:4100–9. 10.1128/AEM.00360-07 17483266 PMC1932762

[77] Choi SH Kim Y Oh S Oh S Chun T Kim SH. Inhibitory effect of skatole (3-methylindole) on enterohemorrhagic Escherichia coli O157:H7 ATCC 43894 biofilm formation mediated by elevated endogenous oxidative stress. Lett Appl Microbiol. (2014) 58:454–61. 10.1111/lam.12212 24372511

[78] Williams BB Van Benschoten AH Cimermancic P Donia MS Zimmermann M Taketani M. Discovery and characterization of gut microbiota decarboxylases that can produce the neurotransmitter tryptamine. Cell Host Microbe. (2014) 16:495–503. 10.1016/j.chom.2014.09.001 25263219 PMC4260654

[79] Jia W Li H Zhao L Nicholson JK. Gut microbiota: a potential new territory for drug targeting. Nat Rev Drug Disc. (2008) 7:123–9. 10.1038/nrd2505 18239669

[80] Mawe GM Hoffman JM. Serotonin signalling in the gut–functions, dysfunctions and therapeutic targets. Nat Rev Gastroenterol Hepatol. (2013) 10:473–86. 10.1038/nrgastro.2013.105 23797870 PMC4048923

[81] Takaki M Mawe GM Barasch JM Gershon MD Gershon MD. Physiological responses of guinea-pig myenteric neurons secondary to the release of endogenous serotonin by tryptamine. Neuroscience. (1985) 16:223–40. 10.1016/0306-4522(85)90059-4 2940472

[82] Lukić I Getselter D Ziv O Oron O Reuveni E Koren O. Antidepressants affect gut microbiota and Ruminococcus flavefaciens is able to abolish their effects on depressive-like behavior. Transl Psychiatry. (2019) 9:133. 10.1038/s41398-019-0466-x 30967529 PMC6456569

[83] Amala K Karthi S Ganesan R Radhakrishnan N Srinivasan K Mostafa AE-ZMA. Bioefficacy of Epaltes divaricata (L.) n-hexane extracts and their major metabolites against the lepidopteran pests spodoptera litura (fab) and dengue mosquito aedes aegypti (Linn). Molecules. (2021) 26:3695. 10.3390/molecules26123695 34204264 PMC8234362

[84] Zeisel SH da Costa K-A. Choline: an essential nutrient for public health. Nutr Rev. (2009) 67:615–23. 10.1111/j.1753-4887.2009.00246.x 19906248 PMC2782876

[85] Craciun S Balskus EP. Microbial conversion of choline to trimethylamine requires a glycyl radical enzyme. Proc Natl Acad Sci U S A. (2012) 109:21307–12. 10.1073/pnas.1215689109 23151509 PMC3535645

[86] Choi YR Kim HS Yoon SJ Lee NY Gupta H Raja G. Nutritional status and diet style affect cognitive function in alcoholic liver disease. Nutrients. (2021) 13:185. 10.3390/nu13010185 33435328 PMC7826807

[87] Holmes E Kinross J Gibson GR Burcelin R Jia W Pettersson S. Therapeutic modulation of microbiota-host metabolic interactions. Sci Transl Med. (2012) 4:137rv6-rv6. 10.1126/scitranslmed.3004244 22674556

[88] Weingarden AR Chen C Bobr A Yao D Lu Y Nelson VM. Microbiota transplantation restores normal fecal bile acid composition in recurrent Clostridium difficile infection. Am J Physiol Gastrointest Liver Physiol. (2014) 306:G310–9. 10.1152/ajpgi.00282.2013 24284963 PMC3920123

[89] Buffie CG Bucci V Stein RR McKenney PT Ling L Gobourne A. Precision microbiome reconstitution restores bile acid mediated resistance to Clostridium difficile. Nature. (2015) 517:205–8. 10.1038/nature13828 25337874 PMC4354891

[90] Kang JD Myers CJ Harris SC Kakiyama G Lee I-K Yun B-S. Bile acid 7α-dehydroxylating gut bacteria secrete antibiotics that inhibit clostridium difficile: role of secondary bile acids. Cell Chem Biol. (2019) 26:27–34. 10.1016/j.chembiol.2018.10.003 30482679 PMC6338514

[91] Steed AL Christophi GP Kaiko GE Sun L Goodwin VM Jain U. The microbial metabolite desaminotyrosine protects from influenza through type I interferon. Science. (2017) 357:498–502. 10.1126/science.aam5336 28774928 PMC5753406

[92] Miki T Goto R Fujimoto M Okada N Hardt WD. The bactericidal lectin RegIIIβ prolongs gut colonization and enteropathy in the streptomycin mouse model for salmonella diarrhea. Cell Host Microbe. (2017) 21:195–207. 10.1016/j.chom.2016.12.008 28111202

[93] Toyosawa T Suzuki M Kodama K Araki S. Highly purified vitamin B2 presents a promising therapeutic strategy for sepsis and septic shock. Infect Immun. (2004) 72:1820–3. 10.1128/IAI.72.3.1820-1823.2004 14977995 PMC356010

[94] Schramm M Wiegmann K Schramm S Gluschko A Herb M Utermöhlen O. Riboflavin (vitamin B2) deficiency impairs NADPH oxidase 2 (Nox2) priming and defense against Listeria monocytogenes. Eur J Immunol. (2014) 44:728–41. 10.1002/eji.201343940 24272050

[95] Hochbaum AI Kolodkin-Gal I Foulston L Kolter R Aizenberg J Losick R. Inhibitory effects of D-amino acids on Staphylococcus aureus biofilm development. J Bacteriol. (2011) 193:5616–22. 10.1128/JB.05534-11 21856845 PMC3187230

[96] Connolly JPR Gabrielsen M Goldstone RJ Grinter R Wang D Cogdell RJ. A highly conserved bacterial D-Serine uptake system links host metabolism and virulence. PLoS Pathog. (2016) 12:e1005359-e. 10.1371/journal.ppat.1005359 26727373 PMC4699771

[97] You JS Yong JH Kim GH Moon S Nam KT Ryu JH. Commensal-derived metabolites govern Vibrio cholerae pathogenesis in host intestine. Microbiome. (2019) 7:132. 10.1186/s40168-019-0746-y 31521198 PMC6744661

[98] Sasabe J Miyoshi Y Rakoff-Nahoum S Zhang T Mita M Davis BM. Interplay between microbial d-amino acids and host d-amino acid oxidase modifies murine mucosal defence and gut microbiota. Nat Microbiol. (2016) 1:16125. 10.1038/nmicrobiol.2016.125 27670111 PMC5074547

[99] Wang Z Klipfell E Bennett BJ Koeth R Levison BS DuGar B. Gut flora metabolism of phosphatidylcholine promotes cardiovascular disease. Nature. (2011) 472:57–63. 10.1038/nature09922 21475195 PMC3086762

[100] Zhu W Gregory JC Org E Buffa JA Gupta N Wang Z. Gut microbial metabolite TMAO enhances platelet hyperreactivity and thrombosis risk. Cell. (2016) 165:111–24. 10.1016/j.cell.2016.02.011 26972052 PMC4862743

[101] Tang WH Wang Z Levison BS Koeth RA Britt EB Fu X. Intestinal microbial metabolism of phosphatidylcholine and cardiovascular risk. N Engl J Med. (2013) 368:1575–84. 10.1056/NEJMoa1109400 23614584 PMC3701945

[102] Koeth RA Lam-Galvez BR Kirsop J Wang Z Levison BS Gu X. l-Carnitine in omnivorous diets induces an atherogenic gut microbial pathway in humans. J Clin Invest. (2019) 129:373–87. 10.1172/JCI94601 30530985 PMC6307959

[103] Li XS Obeid S Wang Z Hazen BJ Li L Wu Y. Trimethyllysine, a trimethylamine N-oxide precursor, provides near- and long-term prognostic value in patients presenting with acute coronary syndromes. Eur Heart J. (2019) 40:2700–9. 10.1093/eurheartj/ehz259 31049589 PMC7963132

[104] Yogarajalakshmi P Venugopal Poonguzhali T Ganesan R Karthi S Senthil-Nathan S Krutmuang P. Toxicological screening of marine red algae Champia parvula (C. Agardh) against the dengue mosquito vector Aedes aegypti (Linn) and its non-toxicity against three beneficial aquatic predators. Aquat Toxicol. (2020) 222:105474. 10.1016/j.aquatox.2020.105474 32259658

[105] Hill MJ. Intestinal flora and endogenous vitamin synthesis. Eur J Cancer Prev. (1997) 6:S43–5. 10.1097/00008469-199703001-00009 9167138

[106] Magnúsdóttir S Ravcheev D de Crécy-Lagard V Thiele I. Systematic genome assessment of B-vitamin biosynthesis suggests co-operation among gut microbes. Front Genet. (2015) 6:148. 10.3389/fgene.2015.00148 25941533 PMC4403557

[107] Aufreiter S Gregory JF Pfeiffer CM Fazili Z Kim YI Marcon N. Folate is absorbed across the colon of adults: evidence from cecal infusion of (13)C-labeled [6S]-5-formyltetrahydrofolic acid. Am J Clin Nutr. (2009) 90:116–23. 10.3945/ajcn.2008.27345 19439459 PMC6443296

[108] Dinan TG Cryan JF. Regulation of the stress response by the gut microbiota: implications for psychoneuroendocrinology. Psychoneuroendocrinology. (2012) 37:1369–78. 10.1016/j.psyneuen.2012.03.007 22483040

[109] Yatsunenko T Rey FE Manary MJ Trehan I Dominguez-Bello MG Contreras M. Human gut microbiome viewed across age and geography. Nature. (2012) 486:222–7. 10.1038/nature11053 22699611 PMC3376388

[110] Karthi S Vasantha-Srinivasan P Ganesan R Ramasamy V Senthil-Nathan S Khater HF. Target activity of isaria tenuipes (hypocreales: clavicipitaceae) fungal strains against dengue vector aedes aegypti (Linn.) and its non-target activity against aquatic predators. J Fungi. (2020) 6:196. 10.3390/jof6040196 33003327 PMC7712577

[111] Macfarlane S Macfarlane GT. Regulation of short-chain fatty acid production. Proc Nutr Soc. (2003) 62:67–72. 10.1079/PNS2002207 12740060

[112] Cummings JH Pomare EW Branch WJ Naylor CP Macfarlane GT. Short chain fatty acids in human large intestine, portal, hepatic and venous blood. Gut. (1987) 28:1221–7. 10.1136/gut.28.10.1221 3678950 PMC1433442

[113] den Besten G van Eunen K Groen AK Venema K Reijngoud DJ Bakker BM. The role of short-chain fatty acids in the interplay between diet, gut microbiota, and host energy metabolism. J Lipid Res. (2013) 54:2325–40. 10.1194/jlr.R036012 23821742 PMC3735932

[114] Smith EA Macfarlane GT. Dissimilatory amino acid metabolism in human colonic bacteria. Anaerobe. (1997) 3:327–37. 10.1006/anae.1997.0121 16887608

[115] Deehan EC Yang C Perez-Muñoz ME Nguyen NK Cheng CC Triador L. Precision microbiome modulation with discrete dietary fiber structures directs short-chain fatty acid production. Cell Host Microbe. (2020) 27:389–404. 10.1016/j.chom.2020.01.006 32004499

[116] Agus A Planchais J Sokol H. Gut microbiota regulation of tryptophan metabolism in health and disease. Cell Host Microbe. (2018) 23:716–24. 10.1016/j.chom.2018.05.003 29902437

[117] Devlin AS Marcobal A Dodd D Nayfach S Plummer N Meyer T. Modulation of a circulating uremic solute via rational genetic manipulation of the gut microbiota. Cell Host Microbe. (2016) 20:709–15. 10.1016/j.chom.2016.10.021 27916477 PMC5159218

[118] Saito Y Sato T Nomoto K Tsuji H. Identification of phenol- and p-cresol-producing intestinal bacteria by using media supplemented with tyrosine and its metabolites. FEMS Microbiol Ecol. (2018) 94:fiy125. 10.1093/femsec/fiy125 29982420 PMC6424909

[119] Zhang LS Davies SS. Microbial metabolism of dietary components to bioactive metabolites: opportunities for new therapeutic interventions. Genome Med. (2016) 8:46. 10.1186/s13073-016-0296-x 27102537 PMC4840492

[120] Yano JM Yu K Donaldson GP Shastri GG Ann P Ma L. Indigenous bacteria from the gut microbiota regulate host serotonin biosynthesis. Cell. (2015) 161:264–76. 10.1016/j.cell.2015.02.047 25860609 PMC4393509

[121] Fung TC Vuong HE Luna CDG Pronovost GN Aleksandrova AA Riley NG. Intestinal serotonin and fluoxetine exposure modulate bacterial colonization in the gut. Nat Microbiol. (2019) 4:2064–73. 10.1038/s41564-019-0540-4 31477894 PMC6879823

[122] Barcik W Wawrzyniak M Akdis CA O'Mahony L. Immune regulation by histamine and histamine-secreting bacteria. Curr Opin Immunol. (2017) 48:108–13. 10.1016/j.coi.2017.08.011 28923468

[123] Valles-Colomer M Falony G Darzi Y Tigchelaar EF Wang J Tito RY. The neuroactive potential of the human gut microbiota in quality of life and depression. Nature Microbiology. (2019) 4:623–32. 10.1038/s41564-018-0337-x 30718848

[124] Koh A Molinaro A Ståhlman M Khan MT Schmidt C Mannerås-Holm L. Microbially produced imidazole propionate impairs insulin signaling through mTORC1. Cell. (2018) 175:947–61. 10.1016/j.cell.2018.09.055 30401435

[125] Maini Rekdal V Bess EN Bisanz JE Turnbaugh PJ Balskus EP. Discovery and inhibition of an interspecies gut bacterial pathway for levodopa metabolism. Science. (2019) 364:eaau6323. 10.1126/science.aau6323 31196984 PMC7745125

[126] van Kessel SP Frye AK El-Gendy AO Castejon M Keshavarzian A van Dijk G. Gut bacterial tyrosine decarboxylases restrict levels of levodopa in the treatment of Parkinson's disease. Nat Commun. (2019) 10:310. 10.1038/s41467-019-08294-y 30659181 PMC6338741

[127] Niranjan-Azadi AM Araz F Patel YA Alachkar N Alqahtani S Cameron AM. Ammonia level and mortality in acute liver failure: a single-center experience. Ann Transplant. (2016) 21:479–83. 10.12659/AOT.898901 27480786

[128] Vince AJ Burridge SM. Ammonia production by intestinal bacteria: the effects of lactose, lactulose and glucose. J Med Microbiol. (1980) 13:177–91. 10.1099/00222615-13-2-177 7381915

[129] Nsenga Kumwimba M Meng F. Roles of ammonia-oxidizing bacteria in improving metabolism and cometabolism of trace organic chemicals in biological wastewater treatment processes: a review. Sci Total Environ. (2019) 659:419–41. 10.1016/j.scitotenv.2018.12.236 31096373

[130] Olde Damink SWM Deutz NEP Dejong CHC Soeters PB Jalan R. Interorgan ammonia metabolism in liver failure. Neurochem Int. (2002) 41:177–88. 10.1016/S0197-0186(02)00040-2 12020618

[131] Liu J Lkhagva E Chung H-J Kim H-J Hong S-T. The pharmabiotic approach to treat hyperammonemia. Nutrients. (2018) 10:140. 10.3390/nu10020140 29382084 PMC5852716

[132] Paster BJ Russell JB Yang CM Chow JM Woese CR Tanner R. Phylogeny of the ammonia-producing ruminal bacteria peptostreptococcus anaerobius, clostridium sticklandii, and Clostridium aminophilum sp. Nov. Int J Syst Bacteriol. (1993) 43:107–10. 10.1099/00207713-43-1-107 8427801

[133] Richardson AJ McKain N Wallace RJ. Ammonia production by human faecal bacteria, and the enumeration, isolation and characterization of bacteria capable of growth on peptides and amino acids. BMC Microbiol. (2013) 13:6. 10.1186/1471-2180-13-6 23312016 PMC3554466

[134] Staley C Weingarden AR Khoruts A Sadowsky MJ. Interaction of gut microbiota with bile acid metabolism and its influence on disease states. Appl Microbiol Biotechnol. (2017) 101:47–64. 10.1007/s00253-016-8006-6 27888332 PMC5203956

[135] Long SL Gahan CGM Joyce SA. Interactions between gut bacteria and bile in health and disease. Mol Aspects Med. (2017) 56:54–65. 10.1016/j.mam.2017.06.002 28602676

[136] Sinal CJ Tohkin M Miyata M Ward JM Lambert G Gonzalez FJ. Targeted disruption of the nuclear receptor FXR/BAR impairs bile acid and lipid homeostasis. Cell. (2000) 102:731–44. 10.1016/S0092-8674(00)00062-3 11030617

[137] Hylemon PB Zhou H Pandak WM Ren S Gil G Dent P. Bile acids as regulatory molecules. J Lipid Res. (2009) 50:1509–20. 10.1194/jlr.R900007-JLR200 19346331 PMC2724047

[138] Copple BL Li T. Pharmacology of bile acid receptors: evolution of bile acids from simple detergents to complex signaling molecules. Pharmacol Res. (2016) 104:9–21. 10.1016/j.phrs.2015.12.007 26706784 PMC4900180

[139] Parséus A Sommer N Sommer F Caesar R Molinaro A Ståhlman M. Microbiota-induced obesity requires farnesoid X receptor. Gut. (2017) 66:429–37. 10.1136/gutjnl-2015-310283 26740296 PMC5534765

[140] Ridlon JM Kang DJ Hylemon PB Bajaj JS. Bile acids and the gut microbiome. Curr Opin Gastroenterol. (2014) 30:332–8. 10.1097/MOG.0000000000000057 24625896 PMC4215539

[141] Mouzaki M Wang AY Bandsma R Comelli EM Arendt BM Zhang L. Bile acids and dysbiosis in non-alcoholic fatty liver disease. PLoS ONE. (2016) 11:e0151829. 10.1371/journal.pone.0151829 27203081 PMC4874546

[142] Urdaneta V Casadesús J. Interactions between Bacteria and Bile Salts in the Gastrointestinal and Hepatobiliary Tracts. Front. Med. (2017) 4:163. 10.3389/fmed.2017.00163 29043249 PMC5632352

[143] Wahlström A Sayin Sama I Marschall H-U Bäckhed F. Intestinal crosstalk between bile acids and microbiota and its impact on host metabolism. Cell Metab. (2016) 24:41–50. 10.1016/j.cmet.2016.05.005 27320064

[144] Ramírez-Pérez O Cruz-Ramón V Chinchilla-López P Méndez-Sánchez N. The role of the gut microbiota in bile acid metabolism. Ann Hepatol. (2017) 16:S21–S6. 10.5604/01.3001.0010.5672 31196631

[145] Chávez-Talavera O Tailleux A Lefebvre P Staels B. Bile acid control of metabolism and inflammation in obesity, Type 2 diabetes, dyslipidemia, and nonalcoholic fatty liver disease. Gastroenterology. (2017) 152:1679–94.e3. 10.1053/j.gastro.2017.01.055 28214524

[146] Wishart DS. Metabolomics for investigating physiological and pathophysiological processes. Physiol Rev. (2019) 99:1819–75. 10.1152/physrev.00035.2018 31434538

[147] Antonarakis SEBALKKWSCRVDLVB. OMMBID: The Online Metabolic & Molecular Bases of Inherited Disease (2005).

[148] Kakiyama G Pandak WM Gillevet PM Hylemon PB Heuman DM Daita K. Modulation of the fecal bile acid profile by gut microbiota in cirrhosis. J Hepatol. (2013) 58:949–55. 10.1016/j.jhep.2013.01.003 23333527 PMC3936319

[149] Grüner N Mattner J. Bile Acids and microbiota: multifaceted and versatile regulators of the liver–gut axis. Int J Mol Sci. (2021) 22:1397. 10.3390/ijms22031397 33573273 PMC7866539

[150] Wishart DS Tzur D Knox C Eisner R Guo AC Young N. HMDB: the human metabolome database. Nucleic Acids Res. (2007) 35:D521–6. 10.1093/nar/gkl923 17202168 PMC1899095

[151] Han J Liu Y Wang R Yang J Ling V Borchers CH. Metabolic profiling of bile acids in human and mouse blood by LC-MS/MS in combination with phospholipid-depletion solid-phase extraction. Anal Chem. (2015) 87:1127–36. 10.1021/ac503816u 25496250

[152] Jäntti SE Kivilompolo M Ohrnberg L Pietiläinen KH Nygren H Orešič M. Quantitative profiling of bile acids in blood, adipose tissue, intestine, and gall bladder samples using ultra high performance liquid chromatography-tandem mass spectrometry. Anal Bioanal Chem. (2014) 406:7799–815. 10.1007/s00216-014-8230-9 25384335

[153] Liu Y Rong Z Xiang D Zhang C Liu D. Detection technologies and metabolic profiling of bile acids: a comprehensive review. Lipids Health Dis. (2018) 17:121. 10.1186/s12944-018-0774-9 29792192 PMC5966875

